# Predicting anticancer hyperfoods with graph convolutional networks

**DOI:** 10.1186/s40246-021-00333-4

**Published:** 2021-06-07

**Authors:** Guadalupe Gonzalez, Shunwang Gong, Ivan Laponogov, Michael Bronstein, Kirill Veselkov

**Affiliations:** 1 Department of Computing, Imperial College London, London, UK; 2 Department of Surgery and Cancer, Imperial College London, London, UK; 3 Institute of Computational Science, University of Lugano (USI), Lugano, Switzerland; 4 Twitter, London, UK; 5 Department of Environmental Health Sciences, Yale School of Public Health, New Haven, CT USA

**Keywords:** Systems biology, Graph deep learning, Genomics, Hyperfoods, Cancer research

## Abstract

**Background:**

Recent efforts in the field of nutritional science have allowed the discovery of disease-beating molecules within foods based on the commonality of bioactive food molecules to FDA-approved drugs. The pioneering work in this field used an unsupervised network propagation algorithm to learn the systemic-wide effect on the human interactome of 1962 FDA-approved drugs and a supervised algorithm to predict anticancer therapeutics using the learned representations. Then, a set of bioactive molecules within foods was fed into the model, which predicted molecules with cancer-beating potential.The employed methodology consisted of disjoint unsupervised feature generation and classification tasks, which can result in sub-optimal learned drug representations with respect to the classification task. Additionally, due to the disjoint nature of the tasks, the employed approach proved cumbersome to optimize, requiring testing of thousands of hyperparameter combinations and significant computational resources.To overcome the technical limitations highlighted above, we represent each drug as a graph (human interactome) with its targets as binary node features on the graph and formulate the problem as a graph classification task. To solve this task, inspired by the success of graph neural networks in graph classification problems, we use an end-to-end graph neural network model operating directly on the graphs, which learns drug representations to optimize model performance in the prediction of anticancer therapeutics.

**Results:**

The proposed model outperforms the baseline approach in the anticancer therapeutic prediction task, achieving an F1 score of 67.99*%*±2.52*%* and an AUPR of 73.91*%*±3.49*%*. It is also shown that the model is able to capture knowledge of biological pathways to predict anticancer molecules based on the molecules’ effects on cancer-related pathways.

**Conclusions:**

We introduce an end-to-end graph convolutional model to predict cancer-beating molecules within food. The introduced model outperforms the existing baseline approach, and shows interpretability, paving the way to the future of a personalized nutritional science approach allowing the development of nutrition strategies for cancer prevention and/or therapeutics.

**Supplementary Information:**

The online version contains supplementary material available at (10.1186/s40246-021-00333-4).

## Background

Cancer is a major healthcare burden and the second leading cause of death in the USA [1]. It has been recently shown, however, that modifying dietary and lifestyle factors alone can prevent between 30 and 40% of all cancer cases [2, 3]. As research continues, it is becoming clearer that nutrition plays a major role in cancer. For example, vegetarian and pescetarian diets have been shown to reduce cancer risk by 10% and 13% respectively [4], and meta-analyses of numerous observational studies have evidenced a reduced risk of colorectal cancer associated to high consumption of whole grains, vegetables, fruit, dairy products, and cruciferous vegetables [5, 6]. However, little is known about the underlying biological mechanisms behind the observed anticancer properties of foods.

Given that understanding such mechanisms is crucial for the design of personalized nutritional strategies for cancer patients, experimental techniques have been used to test anticancer properties of individual molecules within foods. As a result, some biologically-active molecules found in plants (phytochemicals) have been shown to regulate processes linked to oxidative stress, chronic inflammation and epigenetic changes, reducing the risk of cancer [7].

These experimental studies have uncovered anticancer properties of individual food molecules, opening the path towards explaining anticancer properties of foods. However, there are hundreds of molecules within a single food, all of them contributing to a food’s overall effect. Therefore, to provide an accurate explanation of the observed anticancer effects of a given food, the anticancer properties of a greater proportion of its constituent molecules should be studied.

However, evaluating anticancer properties of a single food molecule using experimental techniques is an expensive process which takes months or even years, hence analyzing anticancer properties of all existing molecules within food is hardly feasible. To overcome this limitation, recent efforts have resulted in the compilation of comprehensive databases of phytochemicals [8, 9] which have facilitated computational studies of food molecules [10–12].

Previous computational studies on foods have explored their interactions with diseases and drugs at the genome level, identifying food-drug relationships [11], and beneficial and harmful food-disease pairs [10]. Despite their novelty, these studies only leverage overlap of gene targets or differentially expressed genes to define food-drug and food-disease relationships. These data sources are sparse, pointing to individual genes rather than gene modules affected. However, drug, disease, and food molecule similarities in the context of cancer can only be fully captured by leveraging dysregulated pathways (gene modules), rather than gene target overlap, in the context of protein-protein interaction (PPI) networks, given the heterogeneity of cancer genotypes [13–15].

To uncover dysregulated pathways from gene targets data as input, a set of methods under the term of *network propagation* has been widely used in cancer research. Network propagation, which has been termed an “amplifier of biological signals,” allows finding dysregulated pathways based on the assumption that genes underlying similar phenotypes tend to interact with one another [16].

Veselkov et al. leveraged this set of methods to provide the first large-scale study on anticancer properties of food molecules. In this work [12], the authors predicted anticancer food molecules based on the commonality of mechanisms of action on PPI networks of food molecules compared to FDA-approved anticancer drugs. To this aim, mechanisms of action of drugs on PPI networks were learned using the network propagation algorithm Random Walk with Restarts (RWR). The resulting drug profiles were fed to a supervised machine learning algorithm trained to classify drugs into anticancer and non-anticancer classes. The trained classifier was then used to predict anticancer molecules within food.

The approach used by Veselkov et al. consisted of disjoint modeling and prediction steps, which can result in sub-optimal learned systemic-wide effects of drugs on the PPI with respect to the prediction task. This also translates into a cumbersome optimization process, requiring testing of thousands of hyperparameter combinations and significant computational resources. Furthermore, the network propagation algorithm used by Veselkov et al. in the modeling step to uncover dysregulated pathways propagates or “convolves” information across the network in an unsupervised fashion, using a pre-defined propagation rule.

An alternative to these pre-defined propagation or convolution operators on graphs are graph neural networks (GNNs), developed as part of the trend of deep learning on graphs [17, 18]. Instead of convolving the information across the graph using a pre-defined propagation rule, GNNs have learnable parameters allowing them to propagate or convolve information across the graph in a way that maximizes the performance of a (un)supervised task. This property has been leveraged in a number of applications in bioinformatics incorporating PPI networks, achieving state-of-the-art performance in many cases [19–21].

Building on the capabilities of GNNs and to overcome the limitations of the method proposed by Veselkov et al., we propose an end-to-end graph neural network model which operates directly on graphs representing drugs and is able to model drugs’ effects on the PPI network conditioned on the classification of anticancer therapeutics. Specifically, we feed PPI networks and binary node features encoding drugs’ targets to a graph convolutional encoder to learn the effect of drugs on the PPI network, resulting in vector representations of drugs. Then, learned representations of drugs are fed to an multilayer perceptron (MLP) for prediction of anticancer class.

We train our model in the dataset introduced by Veselkov et al. and compare the performance with their proposed method. Empirical results show that our approach achieves comparative performance in terms of balanced accuracy and significantly higher performance in terms of precision-recall and F1 score, which better capture the performance of a classifier in the case of a highly-imbalanced dataset, such as the one used in this study. Additionally, we offer a causal interpretation of the neural network decision using attribution methods to assign scores to input features.

The major contributions of our work are as follows: ∙ We propose an end-to-end model for the anticancer molecule prediction task, framing the problem as a graph classification task and proposing a graph-based neural network model to solve it ∙ We explore graph pooling based on biological pathways as a way of integrating prior biological information into the neural network architecture ∙ We offer causal interpretation of the neural network which evidences that predictions are made based on biological knowledge

## Methods

### Dataset

A human protein-encoding gene-gene network was compiled using data from STRING v10.5 [22], UniProt (Jan, 2019) [23], COSMIC (Jan, 2019) [24], and NCBI Gene (Jan, 2019) [25]. The subset of experimentally validated protein-protein interactions was selected from STRING. We removed isolated nodes and kept the biggest connected component (15,135 nodes and 177,848 edges).

Information on clinically approved drugs was extracted from DrugBank v5 [26] and DrugCentral (Feb, 2019) [27]. Food molecules were extracted from FooDB (N = 7,793. Nov, 2018) [8]. Drug- and food molecule-gene encoded protein interactions were extracted from STITCH (Jan, 2019) [28].

We extracted pathways from the Kyoto Encyclopedia of Genes (KEGG [29], downloaded version 7.1 from MSigDB [30]). The pathway assignation matrix $ \mathbf {P} \in \mathbb {Z}_{2}^{npathways \times 15,135} $, where *npathways*=186 is an assignation matrix with *P*_*ij*_=1 if gene *j* is involved in pathway *i* and 0 otherwise. Out of the 15,135 genes in the PPI network, 4590 genes had at least a pathway assigned.

Each drug (food molecule) *i* is represented by a graph $G = (\mathcal {V},E)$ of protein-protein interactions, with $|\mathcal {V}| = 15,135$ nodes and |*E*|=177,848 edges, and a feature vector $\mathbf {x}_{i} \in \mathbb {Z}_{2}^{|\mathcal {V}| }$, i.e., one binary feature per node: 1 if the gene is a drug (food molecule) target, 0 otherwise. Hence, we identify drug molecules using only their protein-coding gene targets given that we aim to model their systemic-wide effects on the PPI, task for which drug target information is sufficient. Our dataset contains 2048 drugs and 7793 food molecules. We followed the procedure in [12] to obtain classification labels for the cancer task (positive/negative: 209/1839 drugs).

### Approach

#### Anticancer hyperfood prediction task

We consider the problem of predicting molecules with anticancer properties in foods based on their similarity to FDA-approved anticancer drugs. Food molecules should be predicted as anticancer if their effect on the human genome is similar to that of anticancer drugs.

**Fig. 1:**
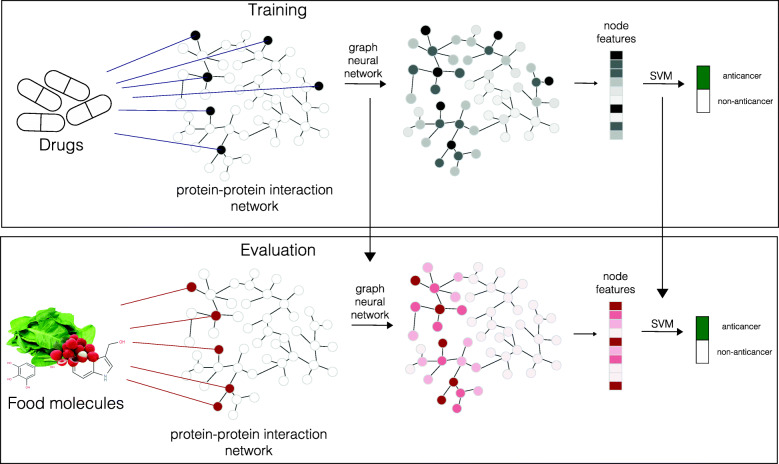
Drug targets are represented as a binary signal on the PPI. We use a GNN to generate a graph embedding representing the systemic-wide effect of the drug on the PPI. We then feed this representation to an MLP for the anticancer prediction task. The model is trained in an end-to-end fashion. After model training, we feed bioactive molecules within foods to the model for the prediction of anticancer food molecules

To this purpose, we build a model to predict anticancer drugs and later use the trained model to predict anticancer food molecules (see Fig. 1). We cast the problem of predicting anticancer drugs as a graph classification task in which drug labels represent whether a drug has been approved to treat cancer (1) or not (0) and our model is trained to output the correct label for each drug. Once the model is trained, it is used to predict anticancer food molecules.

Drugs are represented by the human PPI and their associated drug targets as a binary signal on the PPI. Given a drug *i* represented as a graph $G = (\mathcal {V},E)$, with $|\mathcal {V}|$ nodes and |*E*| edges, and feature vector $\mathbf {x}_{i} \in \mathbb {Z}_{2}^{|\mathcal {V}| }$ (i.e., one feature per node), our aim is to classify it into anticancer/non-anticancer categories. To this aim, we use a non-linear, multi-layer convolutional graph neural network model that operates directly on a graph *G*. We next describe our model for anticancer therapeutic prediction.

### Graph classification model

Our graph classification model takes as input the PPI graph *G* and feature vector **x**_*i*_ of drug *i*, and outputs a classification label indicating anticancer/non-anticancer category. It is comprised by 2 steps. First, a vector representation of the graph *G* is computed using a graph encoder. Then, this representation is fed to a multi-layer perceptron (MLP) which outputs a probability distribution for anticancer/non-anticancer categories for classification (see Fig. 1).

#### Graph encoder

To generate a vector representation of an input graph, we rely on techniques developed as part of the trend of deep learning on graphs [17, 18]. Specifically, we use GNNs, operators that learn how to transform and propagate information, captured by node feature vectors, across the graph. We test three variants of GNNs and compare their performance: GCN [31], GraphSAGE [18], and ChebNet [32].

#### GCN

For a given node, a GCN aggregates transformed feature vectors of its first-order neighborhood. This operator is applied to all nodes in the graph, with transformations applied to neighboring nodes being shared across all nodes.

This is formulated for all nodes in matrix notation as:

$$\mathbf{H}^{(l+1)}_{i} = \sigma\left(\tilde{\mathbf{D}}^{-1/2}\tilde{\mathbf{A}}\tilde{\mathbf{D}}^{-1/2} \mathbf{H}^{(l)}_{i} \mathbf{W}^{(l)}\right) $$

where $\tilde {\mathbf {A}} = \mathbf {A} + \mathbf {I}_{N}$ is the adjacency matrix of the undirected graph *G* with added self-connections, **I**_*N*_ is the identity matrix, $\tilde {\mathbf {D}}$ is the diagonal degree matrix of $\tilde {\mathbf {A}} $, with $\tilde {D}_{ii} = \sum _{j} \tilde {A}_{ij}$. $\mathbf {W}^{(l)} \in \mathbb {R}^{d_{l} \times d_{l+1}}$ is a layer-specific trainable weight matrix and *σ*(·) denotes an activation function such as the rectified linear unit: *ReLU*(·)=*max*(0,·). $\mathbf {H}^{(l)}_{i} \in \mathbb {R}^{|\mathcal {V}| \times d_{l}}$ is the hidden state of drug *i* in layer *l* represented by $|\mathcal {V}|$ nodes and *d*_*l*_ features.

Deeper models, convolving information across the Kth order neighborhood (i.e., embedding of a node depends on all the nodes that are at most K steps away) can be built by stacking K of these layers.

#### GraphSAGE

The GraphSAGE framework learns a function that generates embeddings by aggregating features from a node’s neighbors. We use the *GCN* variant of GraphSAGE which has the form:

$$\hat{\mathbf{H}}^{(l+1)}_{i} = \tilde{\mathbf{D}}^{-1}\tilde{\mathbf{A}} \ \mathbf{H}^{(l)}_{i} \mathbf{W}^{(l)} $$

$$\mathbf{H}^{(l+1)}_{i} = \sigma\left(\frac{\hat{\mathbf{H}}^{(l+1)}_{i}}{\|\hat{\mathbf{H}}^{(l+1)}_{i}\|_{2}}\right) $$

where $\tilde {\mathbf {A}} $ is the adjacency matrix of the undirected graph *G* with added self-connections, $\tilde {\mathbf {D}}$ is the diagonal degree matrix of $\tilde {\mathbf {A}} $, $\mathbf {W}^{(l)} \in \mathbb {R}^{d_{l} \times d_{l+1}}$ is a layer-specific trainable weight matrix, and *σ*(·) denotes an activation function. $\mathbf {H}^{(l)}_{i} \in \mathbb {R}^{|\mathcal {V}| \times d_{l}}$ is the hidden state of drug *i* in layer *l* represented by $|\mathcal {V}|$ nodes and *d*_*l*_ features.

Just like with GCNs, stacking K GraphSAGE layers results in the convolution of information across the Kth order neighborhood.

#### ChebNet

ChebNet is a formulation of convolutional neural networks in the context of spectral graph theory. It relies on the definition of Fourier basis of graphs to define a convolutional filter as a multiplication in the spectral domain. After parametrizing filters using Chebyshev polynomials, a convolutional layer has the form:

$$\mathbf{H}_{i}^{(l+1)} = \sigma\left(\sum_{n=0}^{N-1} \mathbf{Y}^{(l)}_{i,n }\ \mathbf{W}^{(l)}_{n} \right) $$

where *N* is the size of the convolutional filter, $\mathbf {W}^{(l)}_{n} \in \mathbb {R}^{d_{l} \times d_{l+1}}$ is a layer-specific trainable weight matrix, *σ*(·) denotes an activation function, and $\mathbf {Y}^{(l)}_{i,n}$ is computed recursively as:

$$\mathbf{Y}_{i,0}^{(l)} = \mathbf{H}_{i}^{(l)} $$

$$\mathbf{Y}_{i,1}^{(l)} = \hat{\mathbf{L}}\ \mathbf{H}_{i}^{(l)} $$

$$\mathbf{Y}_{i,n}^{(l)} = 2 \ \hat{\mathbf{L}}\ \mathbf{Y}_{i, n-1}^{(l)} - \mathbf{Y}^{(l)}_{i, n-2} $$

$\mathbf {H}^{(l)}_{i} \in \mathbb {R}^{|\mathcal {V}| \times d_{l}}$ is the hidden state of drug *i* in layer *l* represented by $|\mathcal {V}|$ nodes and *d*_*l*_ features. $\hat {\mathbf {L}}$ denotes the scaled and normalized Laplacian $\frac {2\mathbf {L}}{\lambda _{max}} - \mathbf {I}$, with the Laplacian **L**=**I**−**D**^−1/2^**AD**^−1/2^, and *λ*_*max*_ being the maximum eigenvalue of the Fourier decomposition of the graph Laplacian **L**.

Intuitively, each layer of a ChebNet convolves node features from 1 to *N* hops away of each node. For detailed justification and derivation of the ChebNet expression, we refer the reader to [32].

#### Final graph representation

To generate the graph representation of drug *i*, we apply K convolutional layers to the initial drug representation **x**_*i*_. Inspired by the Jumping Knowledge framework [33], the final representation of drug *i* is obtained by concatenating representations generated by all layers:

$$ {\mathbf{Z}}_i=\left[{\mathbf{H}}_i^{(1)}|{\mathbf{H}}_i^{(2)}|\kern1em \dots \kern1em |{\mathbf{H}}_i^{(K)}\right]\in {\mathbb{R}}^{\mid V\mid \times \left({d}_1+{d}_2+\dots +{d}_K\right)} $$

with the input of the first layer being the drug feature vector $\mathbf {H}_{i}^{(0)} = \mathbf {x}_{i} \in \mathbb {R}^{|\mathcal {V}| \times 1}$.

We then feed the graph embedding **Z**_*i*_ generated by the convolutional layers to a fully connected layer to reduce the dimensionality of drug representations by aggregating node features to a single dimension:

$$\mathbf{z}_{i} = \mathbf{Z}_{i} \mathbf{W}_{fc} $$

$\mathbf {z}_{i} \in \mathbb {R}^{|\mathcal {V}| \times 1}$ is the transformed representation of drug *i* and $\mathbf {W}_{fc} \in \mathbb {R}^{\left (d_{1} + d_{2} +... + d_{K}\right) \times 1}$ is a matrix with weights learned to aggregate node feature vectors.

#### Pathway pooling

We consider an alternative architecture in which we introduce pathway pooling, pooling genes in the graph according to biological pathways. Formally, given the graph representation **Z**_*i*_, pathway pooling can be formulated as:

$$\hat{\mathbf{Z}}_{i} = \mathbf{P} \mathbf{Z}_{i} $$

where $\mathbf {P} \in \mathbb {R}^{npathways \times |V|}$ is an assignation matrix with *P*_*ij*_=1 if gene *j* is involved in pathway *i* and 0 otherwise. We then feed the graph embedding $\hat {\mathbf {Z}}_{i} $ to a fully connected layer to reduce the dimensionality of drug representations by aggregating node features to a single dimension:

$$\hat{\mathbf{z}}_{i} = \hat{\mathbf{Z}}_{i} \mathbf{W}_{fcp} $$

$\hat {\mathbf {z}}_{i} \in \mathbb {R}^{npathways \times 1}$ is the transformed representation of drug *i* and $\mathbf {W}_{fcp} \in \mathbb {R}^{\left (d_{1} + d_{2} +... + d_{K}\right) \times 1}$ is a matrix with weights learned to aggregate node feature vectors.

#### MLP

Transformed representation of drugs **z**_*i*_ (or $\hat {\mathbf {z}}_{i}$) are then fed to a 2-layer MLP to output a probability distribution for the classification task:

$$\mathbf{p}_{i} = Softmax\left(ReLU\left(\mathbf{z}_{i}^{T} \mathbf{W}_{l1} + \mathbf{b}_{1}\right) \mathbf{W}_{l2} + \mathbf{b}_{2} \right) $$

where **W**_*l*1_, **b**_1_, **W**_*l*2_ and **b**_2_ are learnable weight matrices. $\mathbf {p}_{i} \in \mathbb {R}^{1 \times 2}$ represents a distribution probability for anticancer/non-anticancer categories for drug *i*.

### Attributing predictions to input features for interpretability

We seek to assess whether the trained model has learned the top biological pathways (i.e., PPI subgraphs) responsible for the anticancer properties of drugs. Given a trained model and an input, an attribution method assigns scores to each input feature that reflect the contribution of that feature to the model prediction. Inspecting the attribution scores reveals what features, in our case, genes were most relevant to the model’s decision. Formally, suppose a function $F : \mathbb {R}^{n} \rightarrow [0, 1]$ represents a deep neural network. The attribution at input $\mathbf {x} =(x_{1},...,x_{n}) \in \mathbb {R}^{n}$ is a vector $A_{F(\mathbf {x})}=(a_{1},...,a_{n}) \in \mathbb {R}^{n}$ where *a*_*j*_ is the contribution of *x*_*j*_ to the prediction *F*(**x**).

In our case, given drug *i* and its feature vector **x**_*i*_ as input, *F*(**x**_*i*_) denotes the probability that the drug belongs to the anticancer category. In the remainder of this section, we will refer to **x**_*i*_ as **x** for notation simplicity. To compute attributions to individual genes, we use the Integrated Gradients method [34]. This method satisfies two fundamental axioms for attribution methods: sensitivity and implementation invariance. For extended definitions and comparisons with other attribution methods, we refer the reader to [34].

This method provides attributions relative to a provided baseline input. Here, we use an input where all drug targets are set to zero. Integrated gradients are defined as the path integral of the gradients along the straightline path from the baseline to the input. The integrated gradient along the *j*^*th*^ dimension for an input **x** and baseline **x**^′^ is defined as follows:

$$a_{j}(\mathbf{x}) ::== \left(x_{j} - x_{j}'\right) \times \int_{\alpha=0}^{1} \frac{\delta F\left(\mathbf{x}' + \alpha \times \left(\mathbf{x} - \mathbf{x}'\right)\right)}{\delta {x}_{j}}\delta \alpha $$

#### Attribution recall score

We would like the attribution scores to recover gene targets that are present in cancer-related pathways. This would mean that our model makes decisions based on feature values in genes relevant for the anticancer properties of drugs. To evaluate the extent to which the model is able to recover cancer-related genes, we introduce a metric called attribution recall score that measures how well the attribution scores recover cancer-related genes. First, we compute attribution scores for samples classified as anticancer. Then, we obtain average attribution scores for samples classified as anticancer across all splits for a given model. With the average attribution scores, we use the PreRanked module of Gene Set Enrichment Analysis (GSEA) [30] to obtain over-represented pathways (from the Kyoto Encyclopedia of Genes- KEGG) in the most positively attributed genes. This measures whether genes in KEGG pathways are overly-present in the most positively-scored genes as compared to what is expected by chance. We then filter over-represented pathways using an FDR of 25% (as advised in GSEA documentation) and measure the recovery of anticancer pathways as the number of over-represented pathways that are related to cancer divided by the total number of anticancer pathways in KEGG.

### Model training

During training, we optimize hyperparameters using cross-entropy loss:

$$- \sum_{i = 1}^{N} \sum_{c = 1}^{2} y_{ic}\ log(p_{ic}) $$

where *y*_*ic*_ is a binary indicator if class label *c* is the correct classification for drug *i* and *p*_*ic*_ is the predicted probability that drug *i* is of class *c* and *N* is the number of drugs in the training set.

We train the model in an end-to-end fashion and optimize all model parameters using back-propagation. We train the model for a maximum of 100 epochs (training iterations) with the Adam optimizer and early stopping with a window size of 20: The training stops if the validation loss does not decrease at least 1*e*^−4^ for 20 consecutive epochs. We implement our model using PyTorch [35] and the Torch Geometric Library [36].

We perform hyperparameter search for the learning rate, l2 regularization on the weights of the neural network, number of dropout layers in the MLP, input data normalization and batch normalization after the convolutional layers.

### Experimental setup

We view the problem of predicting anticancer therapeutics as a graph classification task. Each drug is represented as a graph and its associated node features. We perform 5-fold cross-validation to assess model performance. In each split, 20% of the data is kept as the test set; from the remaining 80%, 10% is used as a validation set to perform early stopping. All splits are generated stratifying samples with respect to labels. It is worth noting that the dataset is highly unbalanced with respect to the target label (only 10.2% of drugs are anticancer). To balance the positive/negative classes, we re-scaled the contribution of each class to the loss function so that it is inversely proportional to class frequencies of each class during training. Models were trained on NVIDIA Tesla V100 and GEForce RTX 2080 GPUs.

We use our models that, for each drug, output a probability of it being an FDA-approved anticancer drug. We evaluate the performance of our presented models against the baseline model introduced in [12]. In this work, the authors represented drug-protein interactions as binary signals on the human PPI network and applied RWR to learn the systemic genome-wide response to the drug intervention. The learned representations were used as input to an SVM for the binary classification task of anticancer/non-anticancer drugs.

To motivate the use of network propagation, we also evaluate versions of the baseline and proposed methods without network propagation. We use an SVM classifier as the counter-part to the baseline method and an MLP as the counter-part of our proposed neural models.

We use various metrics for the comparative analysis of performance. Balanced accuracy is the average of recall obtained on each class; F1 is the weighted average of precision and recall for the positive (anticancer) class; and AUPR is the area under the precision-recall curve and represents the average precision across all recall values.

Hyperparameter settings for every method are determined using a validation set with a grid search over candidate hyperparameter values. For the baseline approach, the grid search for the restart probability is [0.001,0.01,0.1,0.2,...,0.9]. For neural models, hyperparameter candidates can be found in Table 1.

**Table 1:** Hyperparameter space searched

Hyperparameter	Space search
Learning rate	5.10^−4^,5.10^−3^
L2-regularization	1.10^−5^,1.10^−4^,5.10^−4^
Number of convolutional layers	1,2,3
Number of dropout layers	1,2
Batch normalization	*True*,*false*
Feature normalization	*True*,*false*
n-hops for ChebNet	2,4,6

All convolutional layers in our model have *d*=8 hidden units. The first prediction layer has 32 hidden units and the final prediction layer has 2 output units. We use a mini-batch size of 16.

## Results

### Computational complexity

The time complexity of the graph neural layers and neural models used can be found in Table 2. The three proposed variants of graph convolutional layers have comparable time complexity, with the complexity of the ChebNet layer additionally depending on the n-hop used for neighborhood aggregation. Training time is expressed as milliseconds per sample per epoch to facilitate the estimation of the total training time the proposed neural models would need for a different dataset.

**Table 2:** Time complexity of neural layers in *O* notation

Layer/model	Time complexity	Layers	Running time (ms)
GCN	*O*(|*E*| *d*_*l*_ *d*_*l*+1_)	1	5
		2	6
		3	7
GraphSAGE	*O*(|*E*| *d*_*l*_ *d*_*l*+1_)	1	3.5
		2	4.5
		3	6
ChebNet	*O*(*N*|*E*| *d*_*l*_ *d*_*l*+1_)	1	4
		2	5
		3	6

Time complexity of neural models expressed in running time per training iteration per sample

### Prediction of anticancer drugs

We compare the performance of our proposed models to the baseline approach in the anticancer drug prediction task. We report in Table 3 results of our experiments. With the anticancer classification task in mind, we compare the performance of the models using the F1 score and AUPR, the metrics of choice when evaluating classifiers on highly imbalanced datasets.

**Table 3:** Summary of results (%) on anticancer drug prediction

Method	ACC	F1	AUPR	Precision ac	Recall ac	Precision non-ac	Recall non-ac
SVM	79.26 ± 4.2	52.12 ± 5.92	53.35 ± 10.97	41.50 ± 6.75	69.12 ± 10.08	96.31 ± 1.06	88.74 ± 3.20
RWR + SVM	81.13 ± 3.79	51.84 ± 5.79	67.43 ± 8.14	38.98 ± 5.38	75.08 ± 6.92	96.90 ± 0.83	86.67 ± 2.37
MLP	80.62 ± 3.81	66.53 ± 5.02	69.05 ± 5.01	69.75 ± 6.74	64.55 ± 8.23	96.02 ± 0.85	96.68 ± 1.30
GCN	80.52 ± 3.33	63.95 ± 3.90	66.45 ± 5.82	63.33 ± 5.72	65.51 ± 7.42	96.08 ± 0.76	95.54 ± 1.38
GraphSAGE	78.27 ± 6.11	59.93 ± 6.53	64.42 ± 9.96	61.04 ± 5.72	61.15 ± 13.48	95.62 ± 1.37	95.38 ± 1.51
ChebNet	**83.46** ±**2.52**	**67.99** ±**2.87**	**73.91** ±**3.49**	**65.46** ±**4.53**	**71.27** ±**5.58**	**96.71** ±**0.59**	**95.65** ±**0.96**
MLP-P	76.72 ± 2.68	54.40 ± 3.56	59.79 ± 7.64	51.67 ± 11.33	60.73 ± 7.81	95.44 ± 0.72	92.72 ± 3.18
GCN-P	78.70 ± 5.36	57.43 ± 7.61	60.03 ± 8.48	52.77 ± 7.69	64.03 ± 11.05	95.83 ± 1.18	93.37 ± 1.72
GraphSAGE-P	77.09 ± 4.18	54.07 ± 4.88	60.55 ± 9.51	48.87 ± 4.06	61.64 ± 9.65	95.53 ± 0.96	92.55 ± 1.95
ChebNet-P	76.10 ± 2.67	55.71 ± 4.46	59.68 ± 9.53	53.72 ± 4.07	57.86 ± 4.96	95.17 ± 0.53	94.35 ± 0.44

*ACC* = balanced accuracy, *F1* = harmonic mean of precision and recall, *AUPR* = area under the precision-recall curve, *ac* = anticancer, *non-ac* = non-anticancer

**Fig. 2:**
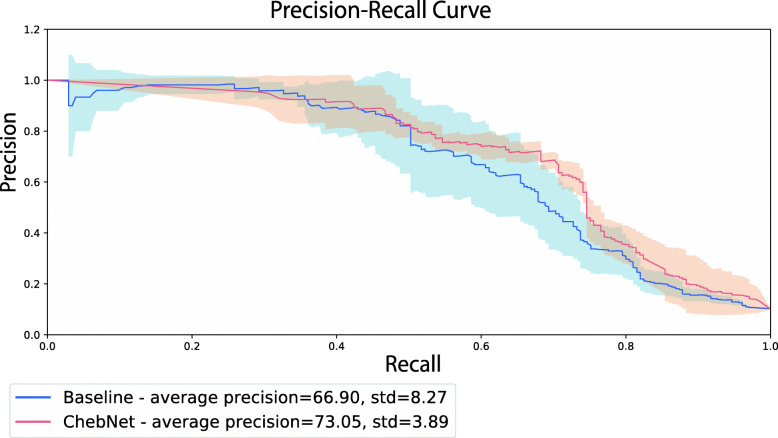
Precision-recall curve of Baseline and ChebNet models across all splits

We see how using a learnable network propagation framework allows our proposed models to outperform the baseline approach by a large margin. The ChebNet variant of our proposed approach had the best performance overall, outperforming the baseline approach by 16.15% (F1) and 6.48% (AUPR). A significantly higher F1 score in the ChebNet model is reflected in that it achieves around 27% higher precision in classification of anticancer samples (with similar recall). The full precision-recall curve, averaged across splits, can be found in Fig. 2, where we can see that the ChebNet model achieves overall higher average precision as compared to the baseline method.

By comparing the AUPR of baseline and neural models with and without the graph structure, a key observation can be drawn that for both, the SVM classifier and the neural models, model performance increases when taking into account the underlying network structure of the data (PPI). Another interesting observation is that introducing pathway pooling translates into a decrease in performance for all models. This could be a result of the incomplete pathway knowledge on the PPI network. The PPI network used in this work has $|\mathcal {V}| = 15,135$ protein-encoding genes with only 4590 genes belonging to at least a pathway (with 186 pathways in the KEGG database). The initial hypothesis was that by having only 30% of genes contributing to the final prediction, the model would increase its performance by focusing only on genes with known biological processes, including cancer processes. However, results indicate that such a hard regularization prevents the model from potentially learning from other equally-relevant but experimentally understudied genes.

### Model interpretation

We compute the attribution recall score for the best-performing model to assess whether the model predicts drugs as anticancer preferentially based on the feature values in cancer-related genes. The attribution recall score for the most positively attributed genes is 85.29*%*. This means that the most positively attributed genes in our model (i.e., the ones whose initial feature value is the most relevant for the prediction task) are preferentially found in cancer-related pathways such that around 85% of cancer-related pathways in KEGG are over-represented. This means that the graph neural model classifies drugs as anticancer preferentially based on the value of the input features in cancer-related genes, which adds to the biological plausibility of the model.

To further validate model’s attributions, we investigate 6 use cases: the top 3 correctly and incorrectly classified drugs as anticancer (i.e., drugs classified as anticancer with highest probability) with experimentally validated pathways in the literature. For each of these drugs **x**_*i*_, we obtain the drug representation computed by our best model **z**_*i*_ and obtain over-represented pathways (FDR <1%) using the PreRanked module in GSEA. We compare over-represented pathways obtained from the drug representations to the knowledge available in the literature on these drugs (see Additional file 1). For all 6 drugs studied, over-represented pathways successfully recovered pathways described in the literature along with cancer-related pathways (see Additional file 1). This means that the representations learned capture the mechanisms of action of drugs.

### Prediction of anticancer food molecules

We compute anticancer likeness of food molecules using the best neural model, with the 5 models trained during the cross-validation and average them to provide a more robust anticancer likeness measure. Of the top predictions (anticancer likeness >= 0.9), 20 molecules were found as additional predictions to those reported in [12] (see Table 4, Additional file 2).

**Table 4:** Anticancer likeness of food molecules was computed using the best-performing neural model

ID	Name	Description
FDB001084	Pancreatin	Digestive enzyme. Used in replacement therapy. Used to prepare protein hydrolysates for pre- and post-operative diets.
FDB006967	Anthracene	Organic compounds containing a system of three linearly fused benzene rings. Anthracene can be found in sorrel. Anthracene is formally rated as an unfounded non-carcinogenic (IARC 3) potentially toxic compound.
FDB008856	2,2’-Bis(4-hydroxyphenyl) propane	Potential food contaminant arising from its use in reusable polycarbonate food containers such as water carboys, baby bottles and kitchen utensils.
FDB011663	Coumestrol	Coumestrol is a natural organic compound in the class of phytochemicals known as coumestans. It has garnered research interest because of its estrogenic activity and its prevalence in some foods, such as soybeans and herbs such as Pueraria mirifica. Coumestrol is a phytoestrogen, mimicking the biological activity of estrogens.
FDB011828	Genistein	Genistein is a phenolic compound belonging to the isoflavonoid group. Isoflavonoids are found mainly in soybean. Genistein and daidzein (an other isoflavonoid) represent the major phytochemicals found in this plant.
FDB012375	Pterostilbene	Pterostilbene is a member of the class of compounds known as stilbenes. Pterostilbene can be found in common grape and grape wine. Pterostilbene is a stilbenoid chemically related to resveratrol.
FDB012974	Mercenene	Found in the common clam Mercenaria mercenaria and Mercenaria campechiensis
FDB014654	Myristicin	Natural organic compound present in the essential oil of nutmeg and to a lesser extent in other spices such as parsley and dill.
FDB016593	2,5-Dihydro-4,5-dimethyl-2-(1-methylpropyl) thiazole	Flavoring ingredient. Reported in hydrolyzed vegetable protein.
FDB020870	1-Methyl-6-phenyl-1H-imidazo[4,5-b]pyridin-2-amine	Food-related mutagen, reported to be the most abundant heterocyclic amine found in cooked meat and fish.
FDB022056	5a-Androstane-3a,17a-diol	Steroid compound.
FDB022182	Isourso-deoxycholic acid	Bile acid.
FDB022318	11alpha-Hydroxy-progesterone	Steroid hormone involved in the female menstrual cycle, pregnancy (supports gestation) and embryogenesis of humans and other species.
FDB023086	Dihydro-testosterone	Potent androgenic metabolite of testosterone.
FDB023772	Testosterone enanthate	Testosterone enanthate is used in androgen substitution.
FDB024072	5b-Dihydro-testosterone	Intermediate in androgen and estrogen metabolism.
FDB028898	Methyl-arsonite	Found in the arsenate detoxification I pathway.
FDB030068	Platinum	Member of the class of compounds known as homogeneous transition metal compounds. Platinum can be found in a number of food items such as white cabbage, sunburst squash (pattypan squash), potato, and broccoli.
FDB030278	17- *α*-hydroxy-pregnenolone	It belongs to gluco/mineralocorticoids, progestogins, and derivatives class of compounds.
FDB030678	Androst-4-en-3,17-dione	It belongs to androgens and derivatives class of compounds.

20 molecules were predicted as additional anticancer molecules to those reported in [12]. Extended description and additional information for each molecule can be found in Additional file 2

We obtained embeddings of these molecules and over-represented pathways from the KEGG and REACTOME databases using GSEA. Over-represented pathways of these molecules captured a wide range of cancer-related mechanisms and signaling pathways including P53 signaling pathway, MAPK signaling pathway, ERBB signaling pathway, and those involved in apoptosis, cell growth, and cell proliferation.

Of the 20 anticancer-predicted molecules, genistein and pterostilbene show the most promise as cancer-preventing agents, as indicated by substantial experimental evidence. Genistein, an isoflavone present in soy, is known to have multiple molecular effects that impact carcinogenesis, such as the inhibition of inflammation, promotion of apoptosis, and modulation of steroidal hormone receptors and metabolic pathways [37]. Therefore, genistein plays an important role in preventing and treating some types of cancer. Pterostilbene, found in grapes and blueberries, is chemically related to resveratrol, a well-studied molecule with antimicrobial, antioxidant, and anti-inflammatory activity which translate into chemopreventive effects [38]. Pterostilbene has shown excellent pharmacological benefits for the prevention and treatment for various types of cancer in their different stages of progression through apoptotic or non-apoptotic anti-cancer activities [39, 40].

## Discussion

The benefit of fruits and vegetables in overall health, and specifically, in cancer, has been well documented through numerous observational studies. However, specific mechanisms of action contributing to the anticancer properties of individual food items are still unknown. This knowledge would facilitate the creation of a personalized nutritional science approach where foods and food supplements could be tailored to individuals based on their particular needs, contributing to their overall health and prevention of cancer.

The first large-scale computational study aiming at analyzing anticancer properties of food molecules was introduced by Veselkov et al. [12]. Here, the authors introduced a machine learning approach to predict food molecules with anticancer properties based on their similitude to FDA-approved anticancer drugs at the genomic level. This approach was comprised of 2 disjoint phases. In the first one, systemic-wide effects of drugs on the genome were learned using unsupervised RWR. In the second, the learned representations were fed to an SVM for the anticancer therapeutic classification task. This can result in sub-optimal learned representations and is tied to a cumbersome optimization process. To address this, we introduced an end-to-end graph neural network model that takes as input a genomic network and binary features representing drugs and food molecules and outputs anticancer classification labels, outperforming the baseline approach by 16.15% (F1) and 6.48% (AUPR).

It is interesting to note the superior performance of the model utilizing the Chebyshev operator. Most operators on graphs, including GCN and GraphSAGE, are designed to generalize across different graphs. In the typical scenario, graph operators learn propagation rules for a dataset composed of samples that each have a different graph, for example, during a drug property prediction task based on drug molecular graphs. These operators are designed with a *message passing* paradigm, in which information from neighboring nodes is transformed and aggregated using a permutation-invariant function. In contrast, the Chebyshev operator includes an element that is characteristic of each graph in its formulation: the graph Laplacian, which allows leveraging the graph eigenvectors to orient the message passing procedure. This might explain the superior performance in our scenario given that all samples share the same graph (PPI), and propagating information in the direction of the graph eigenvectors might result in a more optimal information propagation rule than using a permutation-invariant message passing rule.

To explore miss-classifications of our best model, we investigate the top 3 miss-classified drugs with higher anticancer probability across all splits: calcitriol, cetrorelix, and estrone sulfate (all predicted anticancer with a probability of 99.99%). Calcitriol is them most potent metabolite of vitamin D in humans. Low levels of vitamin D have been consistently associated with an increased risk of colorectal [41–44], breast [44], pancreatic [43, 45], thyroid [46], prostate cancer [47], and cancers of the gastrointestinal tract [43]. Given that calcitriol is the most potent metabolite of vitamin D, calcitriol has been studied as a supplement to address vitamin D deficiency, which has resulted in anticancer properties documented for calcitriol [41, 42, 44, 47].

Cetrorelix, a man-made hormone that blocks the effects of gonadotropin-releasing hormone, has been consistently found to have anticancer properties against ovarian [48, 49], prostate [50, 51], and endometrial cancer [49]. In contrast with calcitriol and cetrorelix, estrone sulfate has been documented to be upregulated in patients with breast cancer, and its inhibition has shown promise as a therapy against breast cancer [52–54]. This highlights the importance of external validation of results given that the model classifies molecules as being similar to anticancer drugs if they act on the genome through similar mechanisms (target similar gene modules). However, under the hypothesis that drugs target gene modules that are altered in cancer patients, molecules predicted as similar to anticancer therapies could represent a cancer-preventing or a cancer-causing molecule.

## Conclusion

We present an approach for predicting anticancer food molecules using a graph convolutional neural network model. The model takes as input a graph structure and signal on the nodes and outputs anticancer likelihood of food molecules. The model outputs a high anticancer likelihood for a given food molecule if said molecule acts on the interactome through similar mechanisms of action as those of FDA-approved anticancer drugs. We show that the graph convolutional model outperforms the baseline model by a significant margin. We also demonstrate that it is able to capture knowledge of biological pathways to predict anticancer molecules based on the molecules’ effects on cancer-related pathways.

The proposed model successfully combines the network propagation and classification tasks, and can be trained in an end-to-end fashion, producing predictions that are based on biological knowledge. This offers a more efficient approach for the anticancer hyperfood prediction task and represent a step forward in paving the way to the future of gastronomic medicine.

## Supplementary Information

**Additional file 1** Analysis of over-represented pathways.

**Additional file 2** Top predicted anticancer food molecules.

## Data Availability

Genome data can be collected from STRING [22] (https://string-db.org), UniProt [23] (https://www.uniprot.org), COSMIC [24] (https://cancer.sanger.ac.uk/cosmic), and NCBI Gene [25] (https://www.ncbi.nlm.nih.gov/gene/). Data to build the pathway assignation matrix can be downloaded from GSEA [30] (https://www.gsea-msigdb.org/gsea/msigdb). Drug data can be extracted from DrugBank [26] (https://www.drugbank.ca), DrugCentral [27] (http://drugcentral.org), and STITCH [28] (http://stitch.embl.de). Food data can be extracted from FooDB [8] (https://foodb.ca) and STITCH [28] (http://stitch.embl.de). The code and data to reproduce our results can be downloaded from GitHub (https://github.com/ggonzalezp/hyperfoods) Declarations
